# Effects of AlN and BCN Thin Film Multilayer Design on the Reaction Time of Ni/Ni-20Cr Thin Film Thermocouples on Thermally Sprayed Al_2_O_3_

**DOI:** 10.3390/s19153414

**Published:** 2019-08-03

**Authors:** Wolfgang Tillmann, David Kokalj, Dominic Stangier, Volker Schöppner, Hatice Malatyali

**Affiliations:** 1 Institute of Materials Engineering, TU Dortmund University, Leonhard-Euler-Straße 2, 44227 Dortmund, Germany; 2 Kunststofftechnik Paderborn, Paderborn University, Warburger Straße 100, 33098 Paderborn, Germany

**Keywords:** nickel-chromium, aluminium nitride, boron carbon nitride, thermovoltage, resistivity, thin film thermocouples, reaction time, physical vapor deposition, multilayer

## Abstract

Thin film thermocouples are widely used for local temperature determinations of surfaces. However, depending on the environment in which they are used, thin film thermocouples need to be covered by a wear or oxidation resistant top layer. With regard to the utilization in wide-slit nozzles for plastic extrusion, Ni/Ni-20Cr thin film thermocouples were manufactured using direct-current (DC) magnetron sputtering combined with Aluminiumnitride (AlN) and Boron-Carbonitride (BCN) thin films. On the one hand, the deposition parameters of the nitride layers were varied to affect the chemical composition and morphology of the AlN and BCN thin films. On the other hand, the position of the nitride layers (below the thermocouple, above the thermocouple, around the thermocouple) was changed. Both factors were investigated concerning the influence on the Seebeck coefficient and the reaction behaviour of the thermocouples. Therefore, the impact of the nitride thin films on the morphology, physical structure, crystallite size, electrical resistance and hardness of the Ni and Ni-20Cr thin films is analysed. The investigations reveal that the Seebeck coefficient is not affected by the different architectures of the thermocouples. Nevertheless, the reaction time of the thermocouples can be significantly improved by adding a thermal conductive top coat over the thin films, whereas the top coat should have a coarse structure and low nitrogen content.

## 1. Introduction

In the future, surface integrated sensor systems will become increasingly significant for smart products. Especially for tribological contacts, a sufficient knowledge concerning the temperature or pressure is of special interest. For example, in order to measure the temperature in combustion engines [1] or in plastic injection moulds [2] a fast response of the thermocouples is necessary. Temperature measurement and control are very important in polymer extrusion, especially to ensure the process stability and quality of the products [3]. For this reason, thin film thermocouples and thin film pressure sensors are ideal for a variety of applications. Thin film thermocouples have a lower mass compared to wire thermocouples and, accordingly, respond faster [4].

For thin film thermocouples, a variety of material combinations are available, whereby the combination depends on both the temperature range as well as the required accuracy. Reported parings in the literature are Pt/Pd, Pt/Pt-Rh, TiC/TaC, Ni/Au, Pt/Ni, Au/Sn, Au/Al, Ni/Fe, Cu-Fe, Cu-Ni, Ni-Cr, Cu-constantan, Fe-constantan and chromel-alumel [2,5,6,7,8,9,10]. The Ni/Ni-20Cr alloy is industrially the most commonly used combination [11,12] and thus, the alloy is used in this study as well. Various approaches to improve the thermoelectric properties, such as the thermal voltage or the response time of thin-film thermocouples have been studied. On the chemical level, it is possible to influence the thermoelectric properties of a thermocouple by varying the chemical composition or by doping. In a previous study [13], Ni/Ni-Cr thermocouples were deposited with 10 at.-%, 20 at.-% and 30 at.-% Cr, whereby the thermovoltage is not influenced significantly. Indeed, the reaction time is the fastest for the Ni/Ni-20Cr thin film thermocouple. By changing the rhodium content, a greater Seebeck coefficient and a higher reproducibility can be achieved for Pt/Pt-Rh thermocouples as reported in Reference [14]. However, in certain cases, it is not possible to change the chemical composition. An approach without changing the composition, is to decrease the sensor size of the thin film thermocouples to foster a faster response time and a higher sensitivity [13,15]. In addition, it is possible to influence the physical structure and the morphology of the thin layers by adjusting the PVD deposition parameters, so that the thermoelectric properties are changed [16]. An increased sputtering power as well as a higher substrate temperature become obvious in a decreased Temperature Coefficient of Resistance as well as the electrical resistance of NiCr thin films with 20 at.-% Cr [17], whereby these properties affect, among others, the applicability for thermoelectric applications. Moreover, due to a higher bias-voltage, a lower electrical resistance can be achieved for Ni-20Cr thin films [16]. Furthermore, it is possible to change the growth behaviour and accordingly, the electrical and physical properties of the thin films by changing the substrate material. For Ni/Ni-20Cr thin films, it is reported that the physical structure changes from a (111) oriented to a polycrystalline film when using a copper substrate instead of a silicon substrate [18]. Simultaneously, the crystallite size increases from 8.8 nm for the Si substrate to 27.8 nm for the Cu substrate. This leads to a change in the electrical resistivity from 1.28 × 10^−4^ Ωcm to 6.69 × 10^−4^ Ωcm.

In order to protect thin-film thermocouples from mechanical and tribological stresses as well as oxidation processes, they can be coated with a hard and wear resistive layer. It is reported, that Pt-Pt-Rh thermocouples are coated with Al_2_O_3_ or AlN for wear protection [8,19]. For Ni/NiCr thin film thermocouples, top coats consisting of HfO_2_ are used as an insulating layer [9], while TiN and Al_2_O_3_ are utilized for wear protection [10,20]. Ni/NiCu thin film thermocouples are sandwiched between Al_2_O_3_ thin films, to isolate the conducting paths from the substrate and to protect the thermocouples during sliding [7]. Kwon et al. also deposited a thin Al_2_O_3_ film prior to the coating of the Ni-20Cr thin film to ensure an insulation between the Ni-20Cr thin film and the silicon substrate [21]. However, an influence of the top coat on the thermoelectric properties has only been rarely investigated. The influence of a AlN and Al_2_O_3_ top coat on the properties of Pt/Pt-Rh thin film thermocouples are analysed in Reference [19]. It is reported that the thermovoltage of the thermocouple coated with AlN is higher up to 1100 K when compared to the thermocouple with Al_2_O_3_. Moreover, the increase of the thermovoltage is more sharply for the thermocouple with an Al_2_O_3_ top coat, whereas the thermovoltage saturates for the other thermocouple with increasing temperatures. 

Thin films of Aluminium nitride (AlN) can be used for a variety of applications, since AlN reveals useful chemical as well as physical properties [22], such as a high hardness and temperature stability [23]. Well grown AlN films feature a high thermal conductivity ranging from 100 W/mK to 320 W/mK [23,24,25,26,27] but, at the same time, expose a great electrical resistivity between 10^10^ Ωcm and 10^17^ Ωcm [23,28]. The mentioned properties are dependent on the structure of the thin films, which can be altered with the deposition parameters [22,24] and the nitrogen content [25]. A low sputtering power leads to a cubic rock salt structure, whereby a high power leads to a hexagonal Wurtzite structure [29]. The thickness, grain size and orientation of the AlN thin films affect the thermal conductivity [25,27,30]. In addition to AlN, boron nitride (BN) is a good choice for thermoelectric applications, since it also features a high thermal conductivity and electrical resistivity [31]. However, pure cubic BN thin films reveal high residual stresses and a low oxidation resistance [32]. The incorporation of C reduces the residual stresses [33] and combines the properties of C and BN [34].

In this study, AlN and BCN thin films are deposited as thermal conductive and electrical isolating top coats on Ni/Ni-20Cr thin film thermocouples, which are synthesized on thermally sprayed Al_2_O_3_, to analyse the influence of the different top coats on the reaction time of the thin film thermocouple. Therefore, the deposition parameters of the top coats were changed in the medium frequency (mf) magnetron sputtering process. BCN is deposited as alternative to AlN since BCN features a different microstructure, which can affect the friction between the melt and the tool surface in the intended application. Moreover, the thermocouples were sandwiched between the mentioned nitride thin films to analyse the effect of the different substrate materials on the growth behaviour of the Ni and Ni-20Cr conducting paths and, accordingly, the thermoelectric properties and the reaction time. To evaluate the reaction time of the different multilayer designs, the step function response provides a possibility to describe the reaction time of the thermocouples. The reaction time is especially important for cyclic processes with large temperature fluctuations. In general, the step function response behaves like an exponential function. Two different characteristic times define the step function [35]:

- half-life period t_50_: time to reach 50% of the temperature jump
- ninety percent time t_90_: time to reach 90% of the temperature jump

## 2. Materials and Methods

### 2.1. Deposition Process

**Figure 1:**
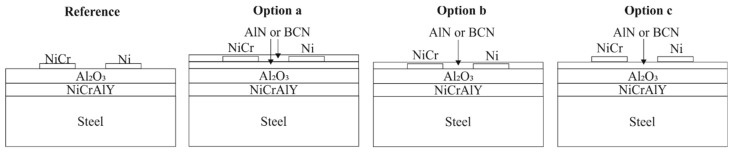
Multilayer design of Ni/NiCr thin film thermocouples with and without nitride top and bottom layers.

A multilayer design was used to produce Ni/NiCr thin film thermocouples. 1.2235 (AISI-L2) steel substrates were selected as substrate material. To ensure an electrical isolation and a thermal insulation between the substrate and the thin film thermocouples, first a NiCrAlY (AMPERIT 413.003, HC Starck, München, Germany) bond coat and subsequently a Al_2_O_3_ (Metco 6062, Sulzer Metco, Pfäffikon, Switzerland) coating were deposited on the steel substrate by means of atmospheric plasma spraying (APS) as reported in Reference [16]. Prior the deposition of the thin films, the Al_2_O_3_ layer were ground and polished to a roughness of Rz = 1.45 ± 0.27 µm. The thin films were synthesized using a magnetron sputtering device type CC800/9 Custom (CemeCon AG, Würselen, Germany). The different multilayer designs are visualized in Figure 1. For the reference thermocouple, the Ni/Ni-20Cr conducting paths were directly dc sputtered on the Al_2_O_3_ coating, using a masking system as reported in Reference [13]. The other designs, labelled Option a, Option b and Option c, consist of the same conducting paths and an additional mf sputtered nitride thin film as a top and/or bottom layer consisting of AlN or BCN.

**Figure 2:**
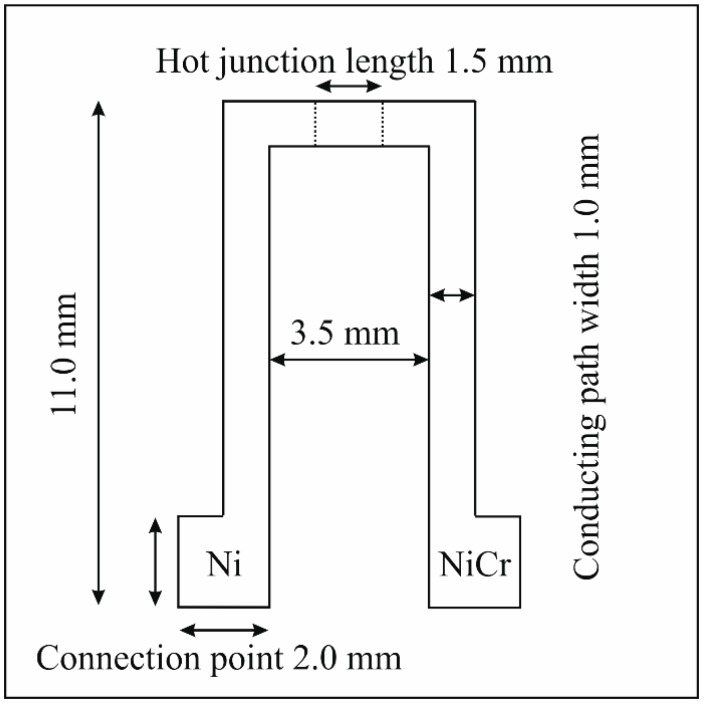
Drawing of the design of the thin film thermocouples with corresponding sizes.

The dimensions of the thin film thermocouples were kept constant for all designs according to Figure 2. As shown in Figure 2, both legs overlap for 1.5 mm at the hot junction point. 

The deposition parameters of the Ni/Ni-20Cr thermocouples as well of the nitride thin films are listed in Table 1. Between each layer, an etching process was conducted, using argon and krypton as process gas as well as a bias-voltage of −650 V in mf mode. In case of the AlN thin films, the heating power and the ratio of the Ar/N_2_ gas flow rate were varied to change the structural properties as well as the morphology. The films were deposited on the entire surface of the substrate for the characterization of the AlN, BCN and Ni as well as Ni-20Cr thin films. Thereby, the thickness of the nitride thin films was set to 1250 ± 70 nm. The thickness of the Ni and Ni-20Cr thin films ranged between 2100 nm and 2300 nm, depending on the layer below. For the thin film thermocouples, the deposition times of all thin films were adjusted to a thickness of approx. 520 nm for the Ni and Ni-20Cr conducting paths and approx. 1200 nm for the AlN and BCN thin films. The thin film thermocouples were synthesized according to the multilayer design shown in Figure 1, resulting in a total of 13 different thin film thermocouples.

### 2.2. Thin Film Characterization

The morphology and topography of the Ni, Ni-20Cr as well AlN and BCN multilayer thin films were analysed using a Scanning Electron Microscope (SEM) type JSM 7001F (Jeol, Tokyo, Japan). By means of Energy-Dispersive X-ray Spectroscopy EDS (Oxford Instruments, Abingdon, UK), the influence of the deposition parameters on the chemical composition of the nitride thin films were investigated. X-ray Diffraction (XRD) was used to investigate the physical structure of the thin films. The structure was obtained by using the diffractometer type D8 Advance (Bruker, Madison, WI, USA), equipped with Cu-Kα radiation (1.5406 Å). The voltage and current were set to 40 kV and 40 mA. To determine the crystallite size of the Ni and Ni-20Cr thin films the (111) reflection was selected, whereby the instrumental broadening was subtracted using the standard reference sample 1976a. The influence of the nitride layers on the hardness H and Young’s modulus E of the Ni and Ni-20Cr thin films were analysed by means of a nanoindenter type G200 (Agilent Technology, Santa Clara, CA, USA). To avoid influences of the surface of the thin films or the substrate material on the hardness measurement, the measuring depth was set between 100 nm and 400 nm. The Poisson’s ratio was assumed to be 0.25 and the measurement was performed as suggested by Oliver and Pharr [36]. The resistivity of the thin films was measured using a multimeter type 34420A (Hewlett Packard, Palo Alto, CA, USA) employing the van der Pauw method and a measuring tip edge with a length of 3 mm.

After producing the thin film thermocouples, the functionality was verified. The so-called Seebeck effect was utilized for temperature measurements with Ni/NiCr thermocouples. In general, the Seebeck effect indicates that a low electric current flows between two different electroconductive materials. The electrons of the materials react differently to temperature changes and produce a measurable thermovoltage. According to Reference [37], the thermovoltage can be calculated as follows:

$$\begin{array}{c} U_{A/B}=S_{A/B}\cdot \left(T_{1}-T_{2}\right)=\;S_{A/B}\cdot \Delta T\\ S_{A/B}=\;\mathrm{Seebeck}\;\mathrm{coefficient};\;U_{A/B}=\;\mathrm{Thermovoltage} \end{array}$$

The thermovoltage increases due to the temperature difference between the hot junction point of the materials and the open end. The difference ΔT is defined by the measured temperature $T_{1}$ and the reference temperature $T_{2}$. For the temperature calculation, the reference temperature has to be known, whereby the implemented cold junction compensation of the measuring system was used. The thermocouples are connected by extending the legs of the thermocouple with Ni/NiCr thermoelectric wires to avoid any influences on the thermovoltage [35]. 

In a first step, a calibration of all thermocouples, using industrial type K sensors, has to be conducted. The calibration ensures a valid relation between the input and output values by means of sensor equations. For the investigations, a silicon oil type Marlotherm SH (Sasol, Marl, Germany), filled in an heated oil container, was used. The temperature of the oil bath was controlled by industrial sensors. The connection points of the sensors were connected with a measurement device type Q. bloxx A104 (Gantner instruments, Schruns, Austria) by means of type K compensation cables. After achieving a constant oil bath temperature (T_oil_) of 60 °C and 120 °C, the hot junction of the sensors was submerged into the oil bath. 60 measurement values were recorded for every steady state condition. Using the software test.commander, the temperature was calculated based on the thermovoltage. Therefore, the cold-junction compensation of the measurement device, revealing room temperature, was used. 

Moreover, for the reaction time calculation, impulsive tests at T_oil_ = 120 °C were carried out, to analyse the influence of the multilayer design on the reaction time in the case of impulsive large temperature differences The step response can be used as quality criterion for thermocouples [35,37]. After reaching a steady state condition of the oil bath, the sensors and two industrial reference sensors, revealing room temperature, were put immediately into the oil bath. The recording of the measurement values starts after the thermocouple insertion and is completed after the measured temperature reaches a stable signal of the target temperature. As target the reaction time t_90_ was used. The reaction time t_90_ is reached when the sensor gains 90% of difference between starting and final temperature. The experimental setup for the measurements is the same as for the calibration.

## 3. Results and Discussion

### 3.1. AlN/BCN Thin Films

#### 3.1.1. Morphology and Physical Structure

**Figure 3:**
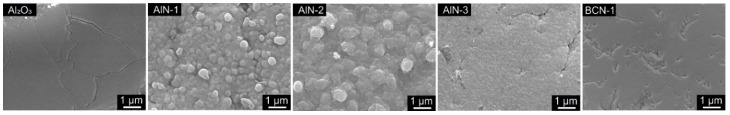
Scanning electron microscope (SEM) images of the topography of the polished Al_2_O_3_ substrate as well as the AlN and BCN thin films.

SEM images of the topography of the polished, thermally sprayed Al_2_O_3_ coating as well as of the topography of the thereon deposited AlN-1–AlN-3 and BCN-1 thin films are shown in Figure 3. The surface of the polished, thermally sprayed Al_2_O_3_ coating reveals a fine structure with a slight crack pattern. The formation of the crack pattern is an inherent process characteristic, due to the rapid solidification of the molten spray particles on the substrate. The topography of AlN-1 and AlN-2 can be compared to a coarse cauliflower-like structure. However, the structure of the AlN-3 thin film is smooth, respectively, fine. The topography of the BCN-1 coating is the finest of the thin films and comparable to that of the polished Al_2_O_3_ topography. The AlN-3 thin film was deposited using a lower nitrogen flow compared with to the other AlN thin films, which is responsible for the change in the topography. Moreover, the substrate temperature, which is affected by the heating power, influences the structure of the topography. The AlN-2 thin film was deposited with the highest temperature (475 ± 7 °C), the AlN-1 film with the second highest temperature (383 ± 4 °C) and the AlN-3 coating with the lowest temperature (325 ± 8 °C), the topography becomes finer in the aforementioned order. For rf (radio frequency) sputtered AlN thin films, it is reported that the structure of the topography is finer due to high nitrogen contents [25]. However, the thin films were only sputtered with nitrogen contents of up to 10% in the chamber, whereby nitrogen contents of 14% and 19% are used for the thin films in this study. Moreover, the growth of thin film depends on the substrate that were used. Since Si was used in the mentioned reference and thermally sprayed Al_2_O_3_ as substrate material in this study, the differences in the influence of the nitrogen content on the topography can be explained. In Reference [38] it is also reported that the physical structure of AlN thin films depends on the substrate material. XRD analyses reveal a polycrystalline hexagonal Wurtzite structure for the AlN thin films. In contrast to that, the BCN thin film features an amorphous structure.

#### 3.1.2. Chemical Composition

The chemical composition of the nitride thin films determined by means of EDS is listed in Table 2. The AlN-1 thin film reveals a nitrogen content of 53.5 ± 0.5 at.-%, while the AlN-2 coating contains a slightly lower nitrogen content of 51.8 ± 0.5 at.-%. However, the change in the nitrogen content is considered to be not significant. As shown by Sharma et al. by means of XPS, a small change in the substrate temperature from room temperature to 100 °C does not lead to a change in the chemical shifts [39]. However, a higher change of the substrate temperature from room temperature to 500 °C alters the chemical shift of the N-1s bonding [39]. The highest nitrogen content of 57.9 ± 0.3 at.-% is measured for the AlN-3 thin film, which is caused by the higher nitrogen content to noble gas ratio in the deposition chamber due to the used gas flow rates. The BCN-1 thin film reveals a boron content of 42.2 ± 2.2 at.-%, a carbon content of 16.8 ± 0.8 at.-% and a nitrogen content of 41.0 ± 2.5 at.-%.

### 3.2. Ni/Ni-20Cr Thin Films

#### 3.2.1. Morphology

**Figure 4:**
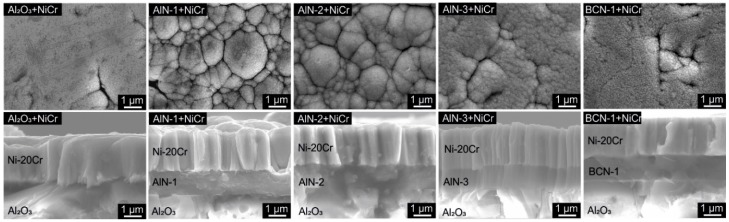
SEM images of the topography (**top**) and cross-section (**bottom**) of Ni-20Cr thin films, deposited on a polished Al_2_O_3_ substrate as well as on AlN and BCN thin films.

Figure 4 shows SEM images of the topography as well as of the morphology of the Ni-20Cr thin films deposited on different substrates. A smooth topography is observed for the Ni-20Cr thin film deposited on the thermally sprayed Al_2_O_3_ coating as well as on the BCN-1 thin film. The topography of the NiCr thin film on AlN-3 is slightly coarser. In contrast to that, a coarse and cauliflower-like topography is observed for the Ni-20Cr thin films synthesized on AlN-1 and AlN-2 thin films. The corresponding cross-sections correlate with the topography. While a fine morphology is observed for the thin films with a smooth topography, a columnar growth is noted for the NiCr thin films with a cauliflower-like topography. When comparing the topography of the Ni-20Cr thin films and of the corresponding substrates, it has to be noted that the growth behaviour of the layer underneath will be adopted by the Ni-20Cr thin films, while a slight coarsening can be observed. A similar growth behaviour for Ni-20Cr thin films is reported in Reference [18]. The NiCr film deposited on copper reveals a higher roughness and a coarser structure compared to the thin film deposited on silicon, since the NiCr thin film grows along the bumps of the rougher copper substrate [18]. 

**Figure 5:**
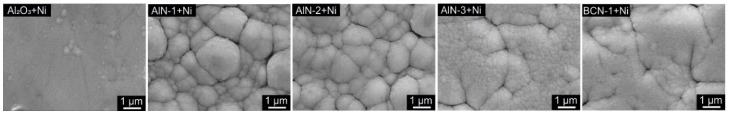
SEM images of the topography (top) of the Ni thin films deposited on a polished Al_2_O_3_ substrate as well as on AlN and BCN thin films.

Corresponding to the SEM images of the topography of the Ni-20Cr thin films, the topography of the Ni thin films on the Al_2_O_3_, AlN and BCN substrates is visualized in Figure 5. It can be observed that the topography of the Ni thin films is comparable to the topography of the Ni-20Cr thin films. Accordingly, regardless of the substrate used, the topography is not affected by adding Cr.

#### 3.2.2. Physical Structure

**Figure 6:**
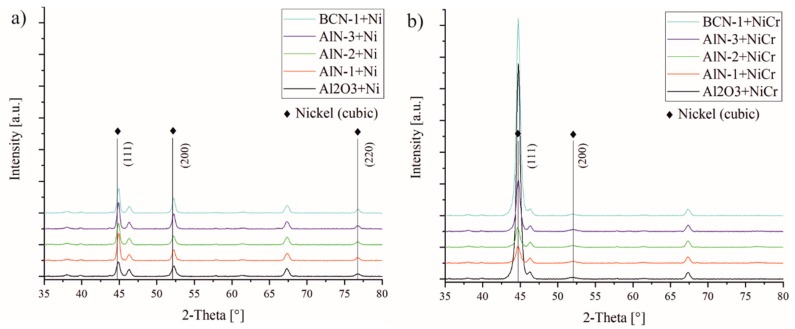
X-ray diffraction (XRD) patterns of the (**a**) Ni and (**b**) Ni-20Cr thin films, deposited on a polished Al_2_O_3_ substrate as well as on AlN and BCN thin films.

The XRD patterns of the Ni and Ni-20Cr thin films, synthesized on different substrates, are shown in Figure 6. A polycrystalline structure, revealing the (111), (200) and (220) reflections, are observed for all Ni thin films. For the (220) reflection, comparable intensities are measured, whereas the (111) and (200) reflection intensities vary for the different substrates. The (111)/(200) intensity ratio increases in the following order: Al_2_O_3_ (1.39), BCN-1 (1.63), AlN-3 (1.75), AlN-2 (2.46) and AlN-1 (2.49). The XRD patterns of the Ni-20Cr thin films (Figure 6b) show only the (111) reflections and a slight shape of the (200) reflections. A more polycrystalline structure of the pure Ni thin film compared to the Ni-20Cr thin film is also reported by Petley et al. [40] for magnetron sputtered coatings. It can be observed that the intensity of the Ni-20Cr (111) reflection decreases in the following order: Al_2_O_3_, BCN-1, AlN-3, AlN-2 and AlN-1. Accordingly, the crystallinity of the Ni-20Cr thin films decreases in the aforementioned order. For the Ni, as well as the Ni-20Cr thin films, no shifts of the reflection positions are noted for the different substrates. Consequently, no changes in the residual stress state of the thin films are to be expected. The other reflections, which are not labelled, belong to the nitride thin films and the thermally sprayed Al_2_O_3_ coating. Moreover, the substrates respectively bottom layers used does not significantly influence the chemical composition of the Ni-20Cr thin films, they reveal a Cr content of 18.2 ± 0.9 at.-% as determined by means of EDS. 

**Figure 7:**
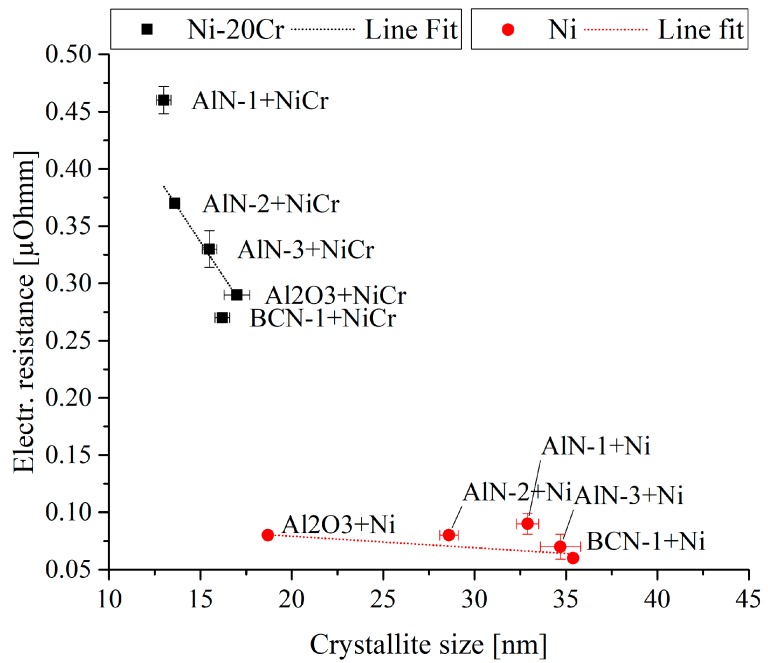
Influence of the substrate layer on the crystallite size and electrical resistance of Ni and Ni-20Cr thin films.

The crystallite size of the Ni and Ni-20Cr thin films are given in Figure 7 in dependence of the substrate layer. First of all, it can be observed that smaller crystallite sizes are obtained for the Ni-20Cr thin films when compared to the Ni thin films. The crystallite size of the Ni-20Cr thin films slightly differs depending on the utilized substrate, ranging from 13.0 ± 0.4 nm (AlN-1) to 17.0 ± 0.7 nm (Al_2_O_3_). For the Ni thin films, crystallite sizes from 18.7 ± 0.2 nm (Al_2_O_3_) to 35.4 ± 0.1 nm (BCN-1) are observed. However, the substrate reveals a greater influence on the crystallite size of the Ni thin films when compared to the Ni-20Cr thin films. The crystallite size of the Ni-20Cr thin films correlates with the topography of the thin films shown in Figure 4, whereby a larger crystallite size is obtained for the thin films with a finer structure. In contrast to that, no dependency is observed for the pure Ni thin films.

#### 3.2.3. Electrical Properties

The dependence of the electrical resistance on the crystallite size of the Ni and Ni-20Cr thin films is visualized in Figure 7. It is observed, that the Ni-20Cr thin films reveal a higher electrical resistance compared to the pure Ni thin films, which is related to the poorer conductivity of chromium compared to nickel. An increase in the electrical resistance with the Cr-content is also observed for rf sputtered Ni-Cr thin films [41]. 

In addition to the composition the electrical resistance depends on the crystallite size of the thin film. The electrical resistance of the Ni-20Cr thin films decreases with an increasing crystallite size from 0.46 ± 0.012 µΩm (AlN-1) to 0.29 ± 0.001 µΩm (Al_2_O_3_). For the Ni thin films, an electrical resistance between 0.08 ± 0.001 µΩm (Al_2_O_3_) and 0.06 ± 0.002 µΩm (BCN-1) is observed. Accordingly, a lower electrical resistance is generally observed for coatings with a larger crystallite size. This can be related to the fact that thin films with a larger crystallite size reveal a smaller amount of grain boundaries, which act as a barrier for the electron transfer [40,42]. Comparing the electrical resistance of both materials, it is observed that the electrical resistance of the Ni thin films is less dependent on the crystallite size when compared to the Ni-20Cr thin films.

For the Ni-20Cr thin films, a dependence of the electrical resistance on the topography is observed. The coatings showing a coarser structure reveal the highest resistivity. The conductivity of a metal can be described with the Boltzmann transport theory [43], whereas the conductivity depends on the scattering time. A shorter scattering time, caused by a high surface roughness or loose and large grain boundaries of a coarse structure, results in a lower conductivity as reported in Reference [18]. In addition, a dependency of the electrical resistance on the intensity of the (111) reflection is observed for Ni-20Cr thin films. With a lower intensity and accordingly a lower crystallinity, a higher electrical resistance is observed as well. In References [44,45] it is also reported that a higher crystallinity leads to a higher electrical conductivity. It can be summarized that the electrical resistance of Ni thin films is lower when compared to Ni-20Cr thin films, independent from the substrate layer used. 

#### 3.2.4. Mechanical Properties

**Figure 8:**
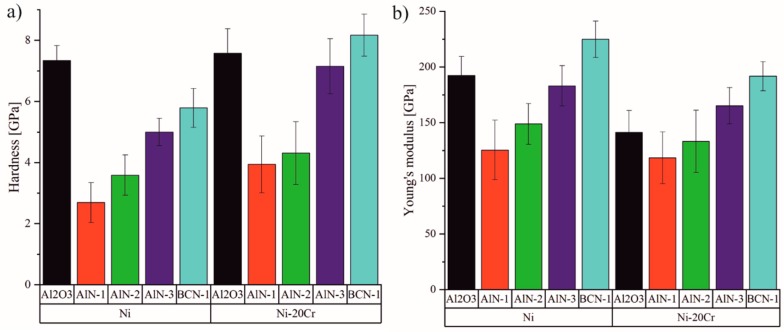
Influence of the substrate layer on the (**a**) hardness and (**b**) Young’s modulus of Ni and Ni-20Cr thin films.

The structural properties of the Ni and Ni-20Cr thin films, which have been modified by using various substrates, are also reflected in the mechanical properties. The thin film thermocouples consist of a multilayer system, which are exposed to high pressures during flat film extrusion. Therefore, the mechanical properties of the individual layers should be high enough to withstand these pressures. The hardness and Young’s modulus of the metallic layers obtained for the different substrates are shown in Figure 8a,b. The hardness of the Ni thin films ranges between 2.7 ± 0.7 GPa and 7.3 ± 0.5 GPa, whereas slightly higher values between 3.9 ± 0.9 GPa and 8.2 ± 0.7 GPa are obtained for the Ni-20Cr thin films. Comparable hardnesses of approx. 5.5 GPa [11] and 7.5 GPa [40] are reported for Ni-20Cr thin films. The higher hardness of the Ni-20Cr thin film compared to the Ni-thin films can be related to the smaller crystallite size of the Cr-containing thin films and the solid solution hardening as also reported in Reference [40]. It has to be mentioned that the hardness of the thin films correlates with the topography and morphology of the thin films. As shown in Figure 4, Ni-20Cr thin films deposited on AlN-1 and AlN-2 thin films show the coarsest structures and the Ni-20Cr thin films grown on Al_2_O_3_ and BCN-1 reveal the finest structure. The lowest hardness values are obtained for the coarse structures whereas the highest hardness values are observed for the finest topographies. The Young’s modulus of the Ni thin films ranges between 125.4 ± 26.7 GPa and 224.8 ± 16.4 GPa. In contrast to the hardness, the Ni-20Cr thin films reveal a lower Young’s modulus between 118.5 ± 23.3 GPa and 191.7 ± 13.1 GPa when compared to pure Ni thin films. 

### 3.3. Ni/Ni-20Cr Thermocouples

#### 3.3.1. Calibration

**Figure 9:**
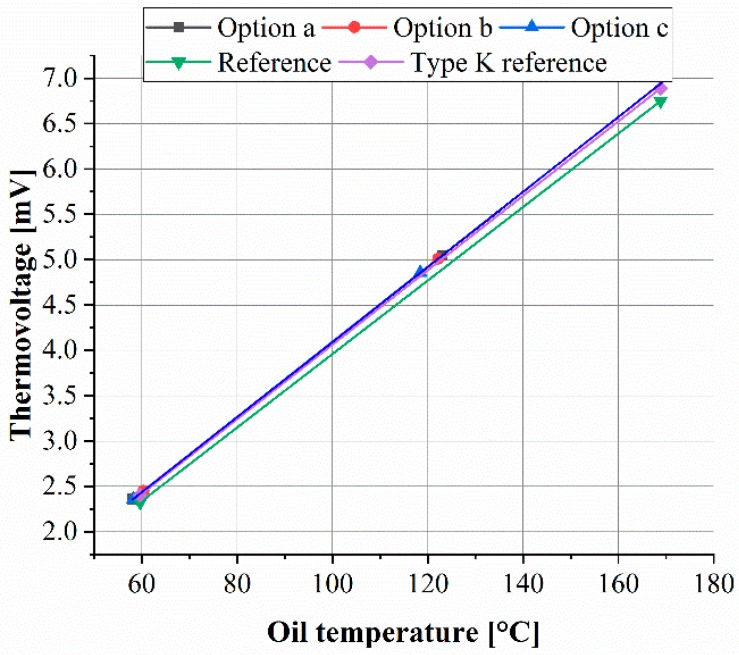
Calibration curve for the Ni/Ni-20Cr thermocouple combined with AlN-1 layers.

The previously analysed thin films were used to deposit thin film thermocouples, whereby one leg consists of the Ni thin film and the other leg of the Ni-20Cr thin film. All thin film sensors were calibrated according to the mentioned experimental setup. An example of the calibration curve, dependence between temperature and thermovoltage, is shown in Figure 9 for the Ni/Ni-20Cr thin thermocouples combined with the AlN-1 nitride thin films. For the thermovoltage, the average values were calculated from the recorded points. Based on the experimental results, a linear correlation can be observed for the thermocouples. The reference thin film thermocouple without a top or intermediate layer reveals the lowest thermovoltage within the analysed temperature range. Changing the thermocouple design by using an AlN-1 intermediate or top layer or both, a higher thermovoltage is observed, whereby the value is slightly higher when compared to the industrial type K reference thermocouple. 

The mathematical equations were calculated from the linear connection of the experimental investigations and are listed in Table 3.

All characteristic curves show no significant differences within the variation of the thin film layers. The Seebeck coefficient of the Ni/Ni-20Cr thin film thermocouple is not influenced by the material, respectively chemical composition, of the additional layer (AlN-1, AlN-2, AlN-3 and BCN-1). There are also no significant differences in the Seebeck coefficients for the different layer designs (a, b and c). All thermocouples reveal a Seebeck coefficient of 0.0414 mV/K, which is comparable to the Seebeck coefficient of 0.0413 mV/K for Ni/NiCr thin film thermocouples as reported by Absar et al. in Reference [46]. Compared to the reference thin film thermocouple without an additional nitride layer with a Seebeck coefficient of 0.0405 mV/K, a slightly higher Seebeck coefficient value is observed. 

**Figure 10:**
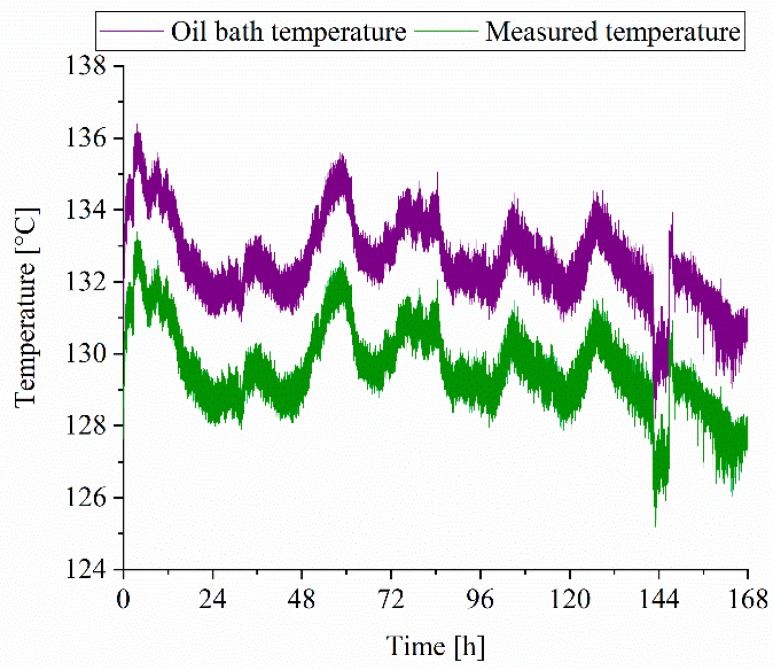
Long-time experiment showing the stability of the reference thin film thermocouple.

The Seebeck coefficient of a given material combination generally depends on the amount of defects, such as dislocations, vacancies or stacking faults [10]. Usually, the Seebeck coefficient of a thin film thermocouple is lower compared to that of a bulk thermocouple since thin films can contain more defects [10]. It is reported for sputtered Ni-Cr thin films that, for example, the electrical resistance and crystallite size change starts during annealing from 250 °C [47,48]. An increase of the thermovoltage and sensitivity is also reported for Pt-Rh/Pt thin film thermocouples when using to vacuum annealing [8]. Since only the thermocouples type a and b were exposed to heat during the deposition process of the nitride thin films and since no change in the Seebeck coefficient occurs, it can be concluded that the thin films reveal a few defects. In addition, this finding is supported by the fact that the Seebeck coefficient of the thin film thermocouples is comparable to that of the wire thermocouples (0.4105 mv/K) used. The drift of the Seebeck coefficient of the reference thin film thermocouple can be attributed to measurement deviations. In addition to the calibration process, the long-time stability on the example of the reference thin film thermocouple was analysed. As to see in Figure 10, the oil bath temperature fluctuates, which can be contributed to the controller of the heating device. The measured temperature of the reference thin film thermocouple follows the temperature of the oil bath and no significant deviations in the difference between the oil bath and measured temperature can be detected after 168 h. Accordingly, the thin film thermocouples reveal a long time stability. The difference between the oil bath and the measured temperature can be related to in homogeneities in the oil bath. 

#### 3.3.2. Reaction Time

**Figure 11:**
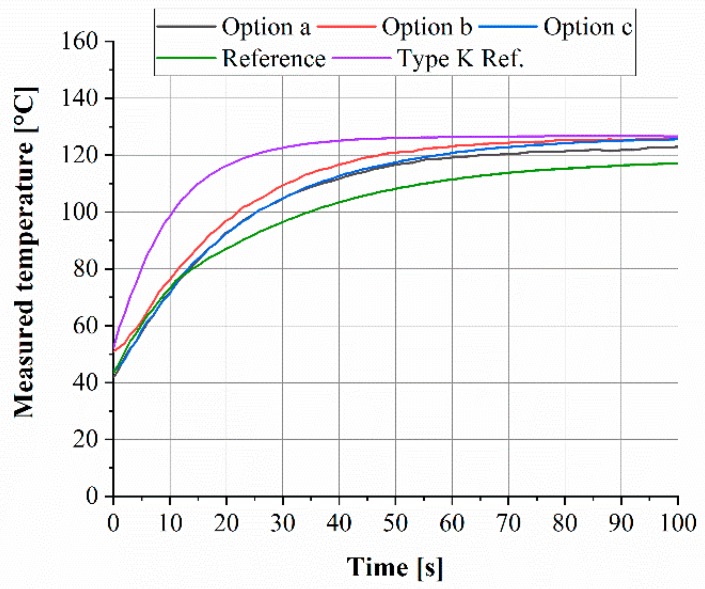
Step function response of the Ni/Ni-20Cr thin film coated with different AlN-1 layer options and step function response of the reference thermocouple.

After the calibration of the thermocouples, impulsive testing was conducted according to the experimental setup. The step function response for the thin film thermocouple coated with different AlN-1 layer options is shown exemplarily in Figure 11.

**Figure 12:**
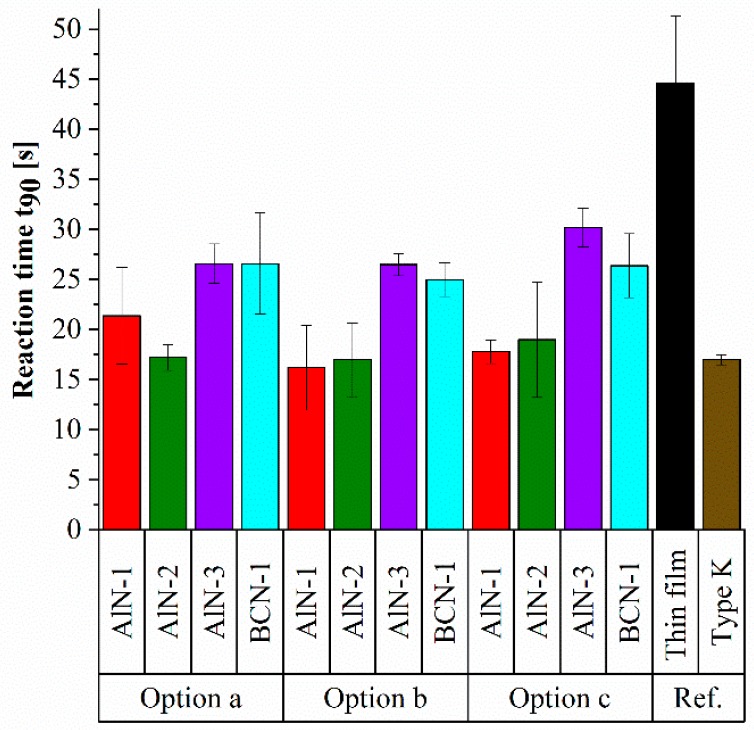
Reaction time of the coated thin film thermocouples and the industrial type K reference.

In the diagram, the temperature is plotted over the time. The step response of each sensor design shows the predicted exponential behaviour. The industrial reference type K thermocouple reveals the fastest step response, while the thin film thermocouples are slightly slower. For a quantitative comparison of the reaction time of all thermocouples, the results are summarized in a bar chart, which is shown in Figure 12. 

Figure 12 shows the reaction times calculated from the step function response of all tested thermocouples. The industrial type K thermocouple needs 17.0 ± 0.5 s for the measurement of a steady state temperature of 120 °C. Compared to that, the reference Ni/Ni-20Cr thin film thermocouple, which is directly deposited on the thermally sprayed Al_2_O_3_ coating, requires a longer time of 44.6 ± 6.7 s to reach the steady state. This extended time can be attributed to the Al_2_O_3_ coating, since the ceramic features a low thermal conductivity. Accordingly, also the ceramic Al_2_O_3_ coating needs to reach the temperature of 120 °C before the thin film thermocouple can reach the steady state. However, the reaction time of the thin film thermocouples can be improved with additional nitride thin film layers. 

One essential point of comparison is the layer material, respectively the composition itself, regardless of the layer design. A significant difference can be observed between the AlN-3 as well as BCN-1 and AlN-1 as well as AlN-2 nitride thin films. While the average reaction time of the thermocouples coated with the first two mentioned layers is 28 s and 26 s, the others need only about 19 s and 18 s to reach a steady state. Within this context, it can be presumed that the composition, respectively morphology of the nitride thin film, has a significant influence on the reaction time of a thermocouple. The AlN-1 and AlN-2 thin film reveal a higher metal/nitrogen ratio when compared to AlN-3 and BCN-1 thin films. Hence, it can be assumed that they reveal a higher thermal conductivity and accordingly enable a faster step response. For Ti(Al)N thin films it is reported that the thermal conductivity depends on the chemical composition and increases with a higher Ti/Al ratio [49]. In addition to the chemical composition, the reaction time of the thin film sensors depends on the morphology of the nitride layers. As shown in Figure 2, the BCN-1 and AlN-3 thin films reveal a finer structure when compared to the other two nitride thin films. A finer structure leads to a higher amount of grain boundaries, which decreases the thermal conductivity due to a higher degree of phonon scattering as reported for TiAlSiN thin films [50].

In addition to the coating material, the different multilayer designs (option a, option b, option c), as described in the experimental setup, have a slight effect on the reaction time. It can be generally observed that the thin film thermocouples with the design option c show in comparison to the other layouts a slightly slower reaction time, whereas options a and b reveal a comparable behaviour within the range of the standard deviation. Concerning the structure, Ni and Ni-20Cr thin films deposited on AlN-1 and AlN-2 thin films reveal the coarsest structure, as shown in Figure 3 and Figure 4. Correlating with the reaction time of the thin film thermocouples with option c, especially the thermocouples deposited on AlN-1 and AlN-2 thin films show the fastest step response. It can be concluded from the experiments that a nitride thin film layer between the Al_2_O_3_ and thin film thermocouples does indeed positively influence the reaction time, since the structure of the thin film thermocouples becomes coarser. Nevertheless, the impact of a nitride top layer is more significant on the reaction time.

## 4. Conclusions

In this study, different multilayer architectures were investigated regarding the impact on the reaction time of Ni/Ni-20Cr thin film thermocouples. Therefore, first of all the influence of different substrate materials on the structural, electrical and mechanical properties of magnetron sputtered Ni and Ni-20Cr thin films was analysed. Both coatings were deposited on thermally sprayed Al_2_O_3_, as well as on a BCN and three different AlN thin films. The crystallite size of the Ni thin films, ranging from 18.7 ± 0.2 nm (Al_2_O_3_) to 35.4 ± 0.1 nm (BCN-1), is larger compared that of Cr containing thin films, ranging from 13.0 ± 0.4 nm (AlN-1) to 17.0 ± 0.7 nm (Al_2_O_3_). With an increased crystallite size, a lower electrical resistance is observed. Moreover, the electrical resistance of the Ni-20Cr thin films is influenced by the topography of the thin films. A coarser structure results in larger grain boundaries, which reduce the conductivity as obtained for the Ni-20Cr thin films, deposited on AlN interlayers. Using different substrates for Ni-20Cr thin films, an electrical resistance between 0.29 ± 0.001 µΩm (Al_2_O_3_) and 0.46 ± 0.012 µΩm (AlN-1) was obtained.

The thin film thermocouple deposited on the thermally sprayed Al_2_O_3_ substrate is ready to use but reveals a longer reaction time, when starting from a cold starting condition, compared to an industrial type K thermocouple. While a top layer of a nitride thin film accelerates the reaction time, an interlayer between the Al_2_O_3_ substrate and Ni/Ni-20Cr thin film thermocouple does not improve the reaction time as much as the top layer. This can be attributed to the changed growth behaviour of the Ni and Ni-20Cr conductive paths deposited on the nitride layers. Another essential result is the determined influence of the different nitride chemical composition and thus, the changed morphology, on the reaction time. While the thermocouples combined with the AlN-3 and BCN-1 layers show a significant longer reaction time, using AlN-1 and AlN-2 layers results in a shorter reaction time, which is comparable to that of the industrial type K thermocouples. The faster step response of the Ni/Ni-20Cr thin film thermocouples combined with AlN-1 and AlN-2 layers can be related to the greater metal content and coarser structure of these nitride thin films. Combining the right layer material with the layer layout, the reaction time of the thin film thermocouples can be reduced from 45 ± 7 s to 16 ± 4 s.

## Figures and Tables

**Table 1:** Deposition parameters of the Ni, Ni-20Cr, AlN and BCN thin films.

Coating	Bias MF [-V]	Target Power [W]	Heating Power [W]	Substrate Temperature [°C]	Chamber Pressure [mPa]	Gas Flow Rate Ratio
Ni	−150	2× 1875 DC (Ni)	1200	260 ± 9	250	Ar/Kr = 2.7
Ni-20Cr	−150	2× 1875 DC (Ni), 1× 1035 DC (Cr)	1200	265 ± 8	250	Ar/Kr = 2.7
AlN-1	−150	2× 3180 MF (Al)	5000	383 ± 4	330	Ar/N_2_ = 6.0
AlN-2	−150	2× 3180 MF (Al)	7000	475 ± 7	330	Ar/N_2_ = 6.0
AlN-3	−150	2× 3180 MF (Al)	3000	325 ± 8	330	Ar/N_2_ = 5.1
BCN-1	−150	1× 3150 MF (B_4_C)	7000	453 ± 3	330	Ar/N_2_ = 26.0

**Table 2:** Chemical composition of Aluminiumnitride (AlN) and Boron-Carbonitride (BCN) thin films.

Composition	AlN-1	AlN-2	AlN-3	BCN-1
Al [at.-%]	46.5 ± 0.5	48.2 ± 0.5	42.1 ± 0.3	-
B [at.-%]	-	-	-	42.2 ± 2.2
C [at.-%]	-	-	-	16.8 ± 0.8
N [at.-%]	53.5 ± 0.5	51.8 ± 0.5	57.9 ± 0.3	41.0 ± 2.5

**Table 3:** Mathematical equation for the correlation between the thermovoltage [mV] (y) and temperature [°C] (x) of the different thin film sensor designs.

Coating	Option a	Option b	Option c
AlN-1	y = 0.0414x − 0.0458	y = 0.0414x − 0.0459	y = 0.0414x − 0.0476
AlN-2	y = 0.0414x − 0.0455	y = 0.0414x − 0.0461	y = 0.0414x − 0.0476
AlN-3	y = 0.0414x − 0.0454	y = 0.0414x − 0.0460	y = 0.0414x − 0.0483
BCN-1	y = 0.0414x − 0.0464	y = 0.0414x − 0.0468	y = 0.0414x − 0.0484
